# Clustering of risk-related modifiable behaviours and their association with overweight and obesity among a large sample of youth in the COMPASS study

**DOI:** 10.1186/s12889-017-4034-0

**Published:** 2017-01-21

**Authors:** Rachel E. Laxer, Ross C. Brownson, Joel A. Dubin, Martin Cooke, Ashok Chaurasia, Scott T. Leatherdale

**Affiliations:** 1 School of Public Health and Health Systems, University of Waterloo, 200 University Avenue West, Waterloo, ON N2L3G1 Canada; 2 Brown School and School of Medicine, Washington University in St. Louis, Campus Box 1196, One Brookings Drive, St. Louis, MO 63130-4899 USA; 3 School of Public Health and Health Systems, Department of Statistics and Actuarial Science, University of Waterloo, 200 University Avenue West, Waterloo, ON N2L3G1 Canada; 4 School of Public Health and Health Systems, Department of Sociology & Legal Studies, University of Waterloo, 200 University Avenue West, Waterloo, ON N2L3G1 Canada

**Keywords:** Obesity, Adolescent, Health promotion, Physical activity, Risk-taking, Latent class analysis, Diet, Behaviour patterns

## Abstract

**Background:**

Canadian youth exhibit a number of risky behaviours, some of which are associated with overweight and obesity. The purpose of this study was to examine the prevalence of 15 modifiable risk behaviours in a large sample of Canadian youth, to identify underlying subgroups based on patterns of health behaviours, and to examine the association between identified subgroups and overweight/obesity.

**Methods:**

Data from 18,587 grades 9–12 students in Year 1 (2012–13) of the COMPASS study and latent class analysis were used to identify patterns and clustering among 15 health behaviours (e.g., physical inactivity, sedentary behaviour, unhealthy eating, substance use). A logistic regression model examined the associations between these clusters and overweight/obesity status.

**Results:**

Four distinct classes were identified: *traditional school athletes, inactive screenagers, health conscious*, and *moderately active substance users*. Each behavioural cluster demonstrated a distinct pattern of behaviours, some with a greater number of risk factors than others. *Traditional school athletes* (odds ratio (OR) 1.15, 95% CI 1.03–1.29), *inactive screenagers* (OR 1.33; 1.19–1.48), and *moderately active substance users* (OR 1.27; 1.14–1.43) were all significantly more likely to be overweight/obese compared to the *health conscious* group.

**Conclusions:**

Four distinct subpopulations of youth were identified based on their patterns of health and risk behaviours. The three clusters demonstrating poorer health behaviour were all at an increased risk of being overweight/obese compared to their somewhat healthier peers. Obesity-related public health interventions and health promotion efforts might be more effective if consideration is given to population segments with certain behavioural patterns, targeting subgroups at greatest risk of overweight or obesity.

## Background

Despite public health efforts, the percentage of children and adolescents that are overweight or obese worldwide has increased dramatically in recent decades, and Canada is no exception [1, 2]. While there is evidence that obesity among children and youth may have reached a plateau [3], the 2015 Senate Report on the state of obesity in Canada still revealed staggering rates of obesity in children aged 5–17 years, with 20 and 12% of children and youth overweight and obese, respectively, triple that of 30 years ago [4]. Mirroring this trend is an increase in chronic conditions (cardiovascular disease, diabetes, stroke, some forms of cancer) traditionally seen among older people, but now observed among children and youth [5].

Adolescence is an important stage of life for the development and maintenance of health and risk behaviours, many of which are associated with overweight and obesity [6, 7]. Several behaviours that have been identified to contribute to increased morbidity and mortality, such as physical inactivity, poor diet, and alcohol, drug, and tobacco use [8] are common among Canadian youth and tend to increase with age [9]. These behaviours do not occur in isolation; rather, evidence suggests that adolescents adopt patterns of healthy or risk behaviours [10] that collectively contribute to poor health outcomes, including overweight and obesity [11, 12]. This is concerning, given that two or more risky health behaviours can amplify the risk of developing chronic diseases [13] and that most youth prevention initiatives are specific to single risk factors (e.g., tobacco control) [14].

While the focus of obesity prevention has shifted from individual to population-level approaches [15] with the intention of reaching individuals at all levels of risk and reducing risk of stigmatization, such broad-based solutions may not be appropriate or effective for all youth [16]. Indeed, there are individual differences in behaviours that are often overlooked in such broad-based interventions [17], which might influence their effectiveness. For example, the majority of school-based obesity prevention programs target two specific sets of health behaviours that are related to obesity-physical activity and dietary behaviours-rarely considering other co-occurring or related health behaviours [13, 18]. More recently, researchers have begun to explore connections between various health behaviours using clustering or latent class analysis, an analytic method that groups heterogeneous populations based on homogeneous characteristics. While these studies have identified behavioural clusters based on patterns of substance use [19], smoking [20], dietary behaviours [21], physical activity patterns [11], or other lifestyle characteristics [22], only a limited number have attempted to draw an association with overweight or obesity [11, 23]. Despite this, all extant studies have confirmed the importance of developing targeted interventions [24], refined to account for heterogeneous characteristics of youth, a population known to exhibit and sometimes adopt a large number of risky behaviours.

The purpose of this study was to (1) examine the prevalence of modifiable risk behaviours in a large sample of Canadian youth, (2) identify homogeneous classes of adolescents based on their obesity-related health and substance use behaviours, and (3) examine how the behavioural classes are associated with overweight/obesity. Identifying the heterogeneity in youth health behaviour patterns might improve both the reach and effectiveness of obesity-related interventions by tailoring programs to those that exhibit behaviours associated with a greater risk of obesity.

## Methods

### Design

COMPASS is a prospective cohort study designed to collect longitudinal data from a sample of secondary school students and the schools that they attend in Ontario and Alberta, Canada [25]. This paper reports on cross-sectional findings from the baseline (Year 1; 2012–13) data collection from 43 purposefully sampled Ontario schools that agreed to use active-information passive-consent parental permission protocols [26]. All student-level data were collected using the COMPASS questionnaire (C_q_). A full description of the COMPASS study and its methods is available online (http://compass.uwaterloo.ca) and in print [25]. The COMPASS study received ethics approval from the University of Waterloo Research Ethics Board, as well as participating school board review panels.

### Measures

#### Health and risk behaviours

Behavioural indicators were selected to represent both theoretically and clinically relevant behaviours associated with overweight and obesity.

##### Physical activity

Four items were used to assess physical activity behaviours. Students recorded (1) time spent in hard (i.e., jogging, team sports) and moderate (i.e., walking, biking to school) physical activity on each of the previous 7 days. Minutes were averaged, and responses were dichotomized to “less than 60 minutes (min) per day” and “more than 60 min per day” to match one component of Canada’s Physical Activity Guidelines for Children and Youth [27]. Students also indicated whether they had participated in (2) physical activities organized by the school (e.g., intramurals, non-competitive clubs) or (3) competitive school sports teams (e.g., junior varsity or varsity sports). For both, students were dichotomized into “participating” or “not participating” in intramurals or varsity sports. Students were asked to record (4) the number of days in the previous week that they had engaged in strengthening exercises. Responses were dichotomized into “3 or more times per week” and “less than three times per week,” as suggested in the Physical Activity Guidelines [27]. Physical activity measures used in COMPASS were found to be both reliable and valid [28].

##### Dietary behaviours

Five items were used to assess dietary behaviours. (1) Breakfast consumption was assessed by asking students if they eat breakfast daily. Students answering “no” to eating breakfast everyday were considered “low breakfast eaters.” (2) Fast food consumption was measured by asking students how many times per week they consumed fast food - those consuming one or more days per week were considered “fast food consumers.” (3) Snacking behaviour was assessed by asking students how many times per week they purchased snacks from a vending machine, corner store, snack bar, or canteen off school property-those purchasing snacks off school property one or more times per week were considered “snackers.” (4) Sugar-sweetened beverage consumption was assessed by asking students how many days, in a usual school week, they drink sugar-sweetened beverages (soda-pop, Kool-Aid, Gatorade, etc.). Those reporting sugar-sweetened beverage consumption three or more days per week were considered “high pop drinkers.” Finally, (5) fruit and vegetable consumption was assessed by asking students to record the number of servings of fruits and vegetables they had eaten the day prior to the survey. Diagrams of Canada’s Food Guide serving sizes were included in the C_q_ for reference [29]. Based on a more conservative estimate of the health benefits of fruit and vegetable consumption, students were dichotomized into those consuming less than five servings and those consuming five or more servings of fruits or vegetables daily [30]. The measure for fruit and vegetable consumption used in COMPASS has been found to be both valid and reliable [31].

##### Sedentary behaviours

Three items were used to assess sedentary behaviour. Students were asked to record how much time per day they usually spent (1) “watching/streaming TV shows or movies,” (2) “playing video/computer games,” and (3) “surfing the internet.” These measures were found to be reliable and valid for use in this sample [28]. Each behaviour was dichotomized into categories of “low” (less than two hours) or “high” (two hours or more), based on the Canadian Sedentary Behaviour Guidelines [32],

##### Substance use behaviours

Three substance use behaviours were included: smoking, marijuana use, and binge drinking. Consistent with previous research, students were classified as (1) smokers if they reported smoking 100 or more cigarettes (in their lifetimes), and smoking at least once in the previous 30 days, or reported using another form of combustible tobacco products (e.g., cigars, cigarillos, roll-your-own tobacco, bidis). (2) Current marijuana users were classified as those who had used marijuana at least once in the last month. (3) Current binge drinkers (i.e., consuming 5 or more drinks on one occasion) were classified as those reporting binge drinking at least once in the last month [18]. Those reporting otherwise were considered non-smokers, non-marijuana users, and non-binge drinkers.

#### Outcome-overweight/obesity

Students’ self-reported height and weight were used to calculate body mass index (BMI). Students were classified as normal weight (combined underweight and normal weight) (corresponding to <24.9 kg/m^2^) or overweight/obese (corresponding to ≥25 kg/m^2^) based on the World Health Organization’s age- and sex-adjusted BMI cut-points [33], and as used in other studies with the same sample of youth [11]. Height and weight measures were validated in a sample of grade 9 students from Ontario, Canada, and both were found to be both highly reliable and valid [31]. Since this study and COMPASS as a project were not meant to be representative, those students with missing BMI data were dropped from the sample.

#### Covariates

Students’ self-reported gender (male, female), grade (9, 10, 11, 12), race (White, Aboriginal (First Nations, Métis, Inuit), other), and weekly spending money ($0, $1–$20, $21–$100, more than $100, “I don’t know”). These were considered covariates based on previous research examining youth health behaviours and BMI [12, 34].

### Statistical analysis

Frequencies for all modifiable health and risk behaviours, demographic information, and outcome measure were examined across the sample.

Latent class analysis (LCA) was used to describe distinct classes, or “clusters” of obesity-related health behaviour patterns and to identify these underlying subgroups based on the combinations of observed behaviours observed. Indicators chosen for the latent class models included the aforementioned 15 health behaviours previously described. LCA uses observed categorical indicators to examine varying groupings and response patterns, and identifies unobserved classes of respondents [35]. Four model selection criteria were used to identify the appropriate number of classes: Akaike information criterion (AIC) [36], Bayesian information criterion (BIC) [37], Consistent Akaike information criterion (CAIC) [38], and adjusted Bayesian information criterion (a-BIC) [38]. Models with 1–6 classes were examined-those with lower values for the model selection criteria are assessed to have a better overall fit to the data [36]. These model selection criteria, combined with model interpretability and posterior probabilities of belonging to a latent class, were used to place participants into the appropriate latent classes. Missing data on individual health behaviours were handled using the expectation-maximization algorithm, and are considered to be missing at random [36]. Given that LCA is a person-centered approach, used to uncover homogeneous groups based on the structure of the data rather than preconceived assumptions of health behaviours and how they might co-occur, results can offer important implications for targeting health promotion strategies to those at greatest risk of overweight and obesity [39].

The association between latent class membership and BMI was examined using a logistic regression model that adjusted for covariates. Analyses considered the clustered nature of the data, and included schools as clusters. All analyses were conducted using SAS 9.4 (SAS Institute, Cary, NC).

## Results

### Study participants

A total of 30,147 students in grades 9 to 12 were enrolled in the 43 COMPASS secondary schools in year 1 (Y1). Overall, 80.2% (*n* = 24,173) of eligible Y1 students completed the C_q_ in class time on the day of the scheduled data collection. Non-responses resulted from student absenteeism (19%), parent refusal (0.9%) or student refusal (0.1%). An additional 5,530 students were missing information on student height, weight (5,274), or other covariates of interest (gender, race, grade, or spending money) and were excluded from the analyses. The final complete case sample included 18,587 students. In comparison to the total sample, the final sample for this study included slightly more males (1.1%), fewer students from grade 9 (2.1%), and slightly more students from grades 11 (1.0%) and 12 (1.6%).

### Participant characteristics

Participant characteristics for the 15 health behaviours examined using LCA are summarized for the total sample in Table 1. Approximately half of the participants were male (51.1%), 73% were white, and over one quarter were overweight or obese (25.6%). Overweight/obesity was found more commonly among males (31%) than females (19.3%).

**Table 1:** Participant characteristics and health behaviours of students participating in Year 1 (2012–13) of the COMPASS Study in Ontario, Canada

	Total % (*n* = 18,587)
Sex (%)	
Male	51.1
Female	48.9
Grade	
9	24.0
10	25.7
11	25.2
12	25.2
Race	
White	73.3
Aboriginal (Off-Reserve)	2.6
Other	24.1
Weekly spending money	
$0	14.7
$1 to $20	30.0
$21 to $100	28.5
More than $100	15.3
I don’t know	11.5
Body Mass Index	
Underweight	1.8
Healthy weight	72.6
Overweight	17.7
Obese	7.9
*Physical activity*	
Physical activity	
< 60 min/day	50.5
Missing (#)	352
Strength training	
≥ 3 days per week	40.2
Missing	153
Participates in school intramurals	
No	59.4
Missing (#)	141
Participates in varsity sports	
No	54.3
Missing (#)	136
*Dietary behaviours*	
Breakfast consumption	
I do not eat breakfast everyday	52.9
Missing	241
Fruit and vegetable consumption	
< 5 servings/day	74.3
Missing	338
Fast food consumption	
≥ 1 time per week	67.5
Missing	443
Snacks purchased off of school property	
≥ 1 time per week	35.2
Missing (#)	508
Sugar sweetened beverage consumption	
≥ 4 days per week	34.8
Missing	482
*Sedentary behaviour*	
Internet Surfing	
≥ 2 h/day	48.8
Missing (#)	16
Video Games	
≥ 2 h/day	29.9
Missing (#)	16
Television	
≥ 2 h/day	52.7
Missing (#)	16
*Other Risky Behaviours*	
Current tobacco user	
Yes	13.9
Current binge drinker	
Yes	25.1
Missing	63
Current marijuana user	
Yes	19.4

### Model fit and selection

Model fit information for models examining 1-6 latent classes is presented in Table 2. A 4-class model was selected as the best-fitting model as it had lower values for each of the model selection criteria, and a more appropriate interpretation than both its smaller and larger counterparts.

**Table 2:** Model fit information for the latent class models, 1–6 classes (*n* = 18,587) from Year 1 of the COMPASS Study in Ontario, Canada (2012–13)

Number of classes	AIC	BIC	CAIC	a-BIC
1	39724.56	39842.01	39857.01	39794.34
2	31636.81	31879.55	31910.55	31781.03
3	23881.12	24249.14	24296.14	24099.78
**4**	**22233.05**	**22526.14**	**22789.35**	**22526.14**
5	20721.96	21340.54	21419.54	21089.49
6	19861.71	20605.61	20700.61	20303.71

*AIC* Akaike information criterion, *BIC* Bayesion information criterion, *CAIC* consistent Akaike information criterion and *a-BIC* adjusted Bayesian information criterion

The latent class model chosen is highlighted in bold

### Class description

**Fig. 1:**
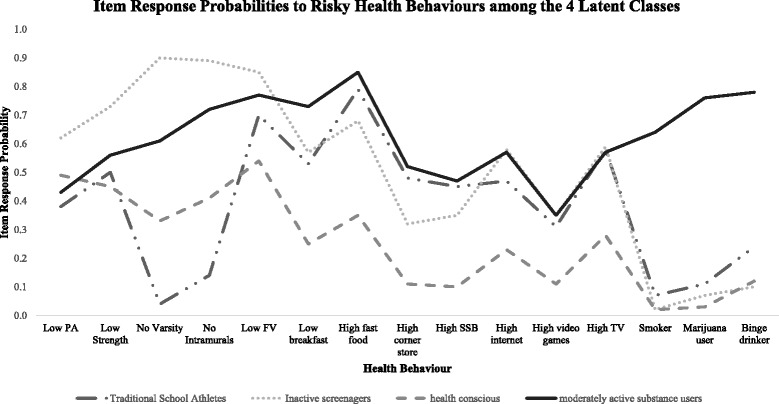
Graphical display of item-response probabilities for health behaviours across the four classes resulting from the LCA in the total sample (*n* = 18,587) from Year 1 (2012–13) of the COMPASS Study in Ontario, Canada. *Health conscious* youth (cluster 3) have the overall healthiest item response probability profile across the latent classes. The *inactive screenagers* and *moderately active substance users* had higher item response probabilities for a larger number of obesity-related and substance use behaviours

The four classes identified in this study, defined by their clustered health behaviours, were named: *traditional school athletes, inactive screenagers, health conscious,* and *moderately active substance users*. Item response probabilities to the health behaviours across the classes are presented graphically in Fig. 1. *Health conscious* youth appeared to have the overall healthiest item response probability profile across the latent classes. The *inactive screenagers* and *moderately active substance users* had higher item response probabilities for a larger number of obesity-related and substance use behaviours.

The first latent class (*traditional school athletes*) included 24% of the sample, and was represented by the highest proportion of youth reporting 60 min of daily physical activity (64%), and of participating in intramural (87%) and varsity sports (99%). Aside from cluster 4 (*moderately active substance users*), a higher proportion of participants in this subgroup were binge drinkers (26%) and marijuana users (9.5%). This subgroup was among the highest in sugar-sweetened beverage consumption (46%), as well as snacking (49%) and fast food consumption (81.5%).

The second latent class (*inactive screenagers*) included 43.3% of the sample, and was characterized by a large number of risky health behaviours-the lowest proportion of youth achieving 60 min of daily physical activity (37%), engaging in strength training at least 3 times per week (26%), and participating in either intramural (11.2%) or varsity sports (8%). Many of the *inactive screenagers* also spent 2 or more hours watching TV (59%), surfing the internet (58%) and playing video games (35%).

The third latent class (*health conscious*), included 16% of the sample, and was characterized by higher physical activity-strength training (59%), intramurals (62%) and varsity sports (73%), the highest proportion of youth consuming breakfast daily (79.5%), and refraining from fast food (71.6%), other snack (93.6%), and sugar-sweetened beverage consumption (93.7%). This subgroup of youth was also the least sedentary, and only few engaged in binge drinking.

Finally, latent class 4 (*moderately active substance users*) included 16.6% of the sample, and was characterized by the highest proportion of youth engaging in risky behaviours: 70% were tobacco users, 79% binge drinkers, and 83% marijuana users. Youth in this subgroup were also the highest consumers of fast food (85%), sugar-sweetened beverages (47%), and snacks from off-school property (52%). While this group reported being moderately active, they were among the highest consumers of screens, with 57% surfing the internet and watching television for two or more hours per day.

### Latent class relations to BMI

There was a significant relationship between the latent classes and BMI (chi-square = 44.39, *p* < 0.0001), with overweight and obesity least represented in the “health conscious” cluster. The highest proportion of overweight/obese youth were in the *moderately active substance users* (28.3%); 26.1% of *inactive screenagers* and 25.6% of *traditional school athletes* were overweight/obese, while only 21.1% of *health conscious* youth were overweight/obese.

### Regression analyses

The association between latent class membership and BMI is presented in Table 3. Participants from the *traditional school athletes* group, the *inactive screenagers*, and the *moderately active substance users* had higher odds of being classified as overweight or obese, compared to those belonging to the *health conscious* group. *The traditional school athletes* were 1.15 (95% CI 1.03–1.29) times more likely to be classified as overweight or obese compared to the healthiest subgroup, while *inactive screenagers* and *moderately active substance users* were 1.33 (95% CI 1.19–1.48) and 1.27 (95% CI 1.14–1.43) times more likely, respectively, to be overweight or obese compared to the *health conscious* group.

**Table 3:** Odds ratio of being overweight/obese according by latent class for the total sample and male and female subsamples from Year 1 (2012–13) of the COMPASS Study in Ontario, Canada

Latent classes	OR (95% CI)
Health Conscious (Latent class 3)	1.00
Traditional School Athletes (Latent class 1)	1.15 (1.03–1.29)^a^
Inactive Screenagers (Latent class 2)	1.33 (1.19–1.48)^b^
Moderately Active Substance Users (Latent class 4)	1.27 (1.14–1.43)^b^

Models were adjusted for grade, gender, race and total spending money

^a^ *p* = 0.0099

^b^ *p* < .0001

## Discussion

This study used latent class and regression analyses to examine patterns of modifiable health behaviours and their association with overweight and obesity in a large sample of youth from Ontario, Canada. The health behaviours and proportion of youth that were overweight or obese in the sample were consistent with other Canadian studies [11, 13]. Results from this study demonstrated four complex combinations of health behaviours among adolescent subgroups, three of which were comprised of students exhibiting poorer health behaviours, increasing their risk of being classified as overweight or obese. Identifying and understanding distinct patterns of health behaviours may help researchers better understand etiological factors of overweight or obesity among youth, and might have important implications for health promotion and public health efforts [14].

A number of studies have investigated the co-occurrence of modifiable behaviours in youth [13, 40, 41], providing insight into the types of behaviours in which youth engage. However, these studies, have been limited as they did not include the mechanism by which particular subgroups of youth engage in similar behavioural patterns. For example, researchers used confirmatory factor analysis to identify an underlying factor for the co-occurrence of behaviours, concluding that a “substance use risk factor” and an “unhealthy eating and sedentary factor” explained youths’ health behaviours. Based on their findings, it might make sense that these factors could be targeted in health behaviour change interventions [40]. However, this might be misleading since the two factors are likely not mutually exclusive-as seen in the present study, substance use behaviours tended to cluster with obesity-related behaviours. As such, cluster techniques such as LCA can provide better insight about patterns of health behaviours, especially those that may not seem intuitively related. One such explanation might include problem-behaviour theory, which suggests an underlying behavioural syndrome drives youth to adopt multiple problem behaviours, possibly caused by an imbalance of risk factors relative to protective factors across personality and socio-environmental domains [42]. Using LCA or analogous clustering methodologies extends the notion that risky behaviours co-occur, but do so in interesting ways that might warrant specific prevention approaches for different risky behaviours in youth.

The literature on overweight and obesity in youth has largely centered around physical inactivity, sedentary behaviour and poor dietary behaviours. Our findings demonstrate that other risky behaviours, including substance use, tend to cluster with these behaviours, suggesting that obesity prevention efforts must move beyond the focus on just physical activity and healthy eating, to include substance use and specific screen-based behaviours [43–45]. In one study, adolescents reporting low levels of physical activity also reported high cigarette smoking, low fruit and vegetable consumption, higher TV watching, failure to wear a seatbelt, and a low perception of academic performance. The authors speculated that intervening on one risky health behaviour might have an effect on reducing other negative health behaviours. To promote healthy behaviours among youth at the critical stage of behavioural development [46] and in an effort to reduce overweight/obesity, it is important to understand optimal behavioural patterns and to place emphasis on strategies that target overall behavioural patterns, rather than single behaviours [14, 47], as well as evaluation studies to investigate their effectiveness.

While *traditional school athletes* were more likely to participate in intramural and varsity sports and to accumulate 60 min of physical activity daily, expectedly, youth in this group were also more likely to binge drink and to use marijuana-considerably more than the *health conscious* and *inactive screenagers*. This makes sense, given the school athlete, or “jock” archetype has often been associated with heavy drinking behaviours [48, 49]. This is similar to previous research by Laska and colleagues, who identified that a “classic” jock subgroup among young adults had the lowest probability of inadequate physical activity, and a higher probability of binge drinking, intoxicated sex, and drunk driving, compared to the other classes [22]. While the *traditional school athletes* were identified as being at greater risk of overweight and obesity than the *health conscious* students, this might be explained by the greater amount of muscle mass often held by athletes, which contributes to a higher BMI-sometimes identifying healthy athletes as overweight or obese. Laska’s research had other similarities, such as a *health conscious* subgroup, characterized by females with favourable diet and physical activity characteristics; however, these females also had the highest probability of unhealthy weight control behaviours [22]. Similar behaviours were found to cluster among university students [50] and adults [51].

Despite the fact that some behavioural clusters were healthier than others, there was a bleak image of the overall health of students in this sample, with all classes exhibiting at least one risky behaviour. This was consistent with national evidence [9]. Fewer than 1 in 5 students in this study belonged to the *health conscious* cluster and were at a lower risk of overweight and obesity. Despite this, even the *health conscious* subgroup, which seemingly had a more favourable behavioural profile, was composed of youth not meeting behavioural recommendations and engaging in risky behaviours. This was consistent with another study, in which subjects in all latent classes exhibited at least one risky behaviour [22]. The risky behaviour found across all four clusters was inadequate fruit and vegetable consumption. This was not surprising; Rossiter and colleagues found that among students in grade 9, only 4% of males and 7% of females were meeting Canada’s Food Guide recommendations for 7–8 servings of fruit and vegetables [52]. This study used a loose interpretation of this Guideline, measuring the proportion of youth consuming a minimum of five servings, as recommended by the CDC [24]. Despite using this lower limit, the proportion of youth adhering to recommendations on fruit and vegetable consumption was still low. This was also not surprising, given that ample research has demonstrated that Canadian adolescents have poor diets [53], including low fruit and vegetable consumption [9], and frequent breakfast skipping and meal consumption away from home [54, 55].

To date, obesity prevention initiatives targeting adolescents have had limited success [16]. This might be because they are school- or community-based, and include all students in order to avoid stigmatization, or because the interventions have targeted a limited number of risky behaviours. These approaches tend to target heterogeneous groups of youth, many of which would not benefit from such interventions. Identifying subgroups could allow researchers to better target formative research and design effective interventions, as well as highlight areas for future research and interventions that can target both broad and specific lifestyle factors. Latent class analysis might allow researchers and public health professionals to tailor interventions that target specific and appropriate subgroups to lead to more refined and effective interventions [12, 56]. This approach can be used to identify groups at highest risk, so that interventions can be more appropriately developed [57]. For example, latent class analysis can provide the evidence required for appropriate audience segmentation for the application of social marketing principles [56]. Future studies can take these analyses further, and refine groups by gender, race, grade, or other non-modifiable characteristics, which may allow for finer and more tailored interventions.

Strengths of this study include the large sample size, high response rate, and the comprehensiveness of the health behaviours examined. This is the first study in Canada or other countries to examine the behavioural clustering of such a large number of behavioural risk factors among youth using latent class analysis, and has included the largest sample size to date in this field. Similar methodologies, such as factor and cluster analyses generate clusters based on empirical rather than theoretical evidence; by pairing the latent class analysis results with model interpretability, our findings provide more substantial evidence of the complexity of youths’ behavioural patterns, thereby better identify high-risk groups for targeted interventions that use integrated approaches accounting for multiple obesity-related health behaviours.

There are several limitations to this manuscript, most notably the use of cross-sectional data, which prevents causal inferences from being made. While many of the behaviours examined in this study have an intuitive causal relationship with overweight/obesity, there are some cases in which being overweight or obesity might increase one’s risk of engaging in risky health behaviours. Longitudinal research, which can be facilitated using the COMPASS study, is needed to follow the outcome of behavioural patterns over time. Second, this study relied on self-reported behaviour and outcome measures, which may be subject to social desirability bias [58]. Although objective measures are ideal, given the sample size, it was not feasible to collect this information using objective measures. And while most of the measures used in this study were found to be reliable and valid, it is possible that the effects in this study were underestimated [50]. However, similar measures of youth behaviours have been appropriate for use in previous studies [13]. Third, there might be other health behaviours found to be associated with BMI in youth; it was not possible to examine all in this study. However, this study included a more comprehensive list of health behaviours than has been used previously. Fourth, although the sedentary behaviour guidelines suggest limiting recreational screen time to a maximum of two hours daily [32], we chose to include each type of screen time individually, dichotomizing each into less than or more than two hours. Had we not, we would have witnessed a ceiling effect, where the majority of the sample was engaging in two or more hours of screen time, thus making it difficult to identify any particular patterns in their health behaviours [13] and underestimating total youth screen time. Fifth, there was some missing information on some of the health behaviours and our latent class analysis assumed these to be missing at random [35], which may have led to a potential misrepresentation of the classes. Less than 2% of students were missing data on any of the behaviours, so this was not likely to be a major problem. Sixth, COMPASS does not collect data on family-level or neighbourhood-level socio-demographics. Similar to the multi-dimensional nature of health behaviours and their co-occurrence, socio-demographic factors at both the family and neighbourhood levels have an influence on health behaviours and health outcomes [59] and would be worth exploring and including in future studies. Finally, clusters and data analyses are driven by the data, and therefore not necessarily generalizable beyond this population. However, the behaviours examined in this study and the behavioural responses of students tend to match those from previous research [11, 57].

Despite these limitations, the findings from this study have important implications for public health and school-based health promotion initiatives. First, although there was limited variability in BMI across the groups, the healthiest cluster still exhibited some unhealthy behaviours, suggesting that all youth, regardless of their health behaviour cluster, might benefit from some level of intervention, to varying degrees. Second, this paper provides further evidence that health behaviours do not occur in isolation, and that a comprehensive approach that considers the clustering of health behaviours is ideal for promoting health behaviours and reducing chronic disease in youth [60]. Such an integrated approach, targeting several risky behaviours, along with ensuring supportive environments within which youth can adopt healthy behaviours, can more likely change the trajectory of children’s health and health behaviours. This can be done through school programs and resources that integrate different aspects of health and well-being. Tailored approaches are more effective and have greater potential of reaching the appropriate audiences than population-based approaches [61]. For example, targeting an obesity-prevention initiative at *traditional school athletes* might focus on reducing binge drinking and marijuana use, rather than focusing on a message to increase physical activity, since the *traditional school athletes* are sufficiently active. This might be achieved through a substance-use policy in schools for athletes, whereby athletes joining sports teams sign a contract and commit their abstinence to substance use and a guarantee to maintain healthy nutrition and reduce their screen time. *Moderately-moving substance* users were those who used several substances and who engaged in other risky behaviours that typically co-occur with substance use. These youth might be best reached by harm reduction and education on substance abuse and poor nutrition, and by reducing the amount of time spent watching TV and surfing the internet. This group might also be reached by promoting intramurals, which might replace some screen time and reduce their risk of engaging in other risky behaviours. Finally, the *inactive screenagers*, demonstrating the lowest physical activity and highest screen time, might be targeted by promoting fun and engaging physical activity opportunities to replace time spent on screens. Increasing access to affordable fruits and vegetables, or creating urban gardens in schools and communities, might increase fruit and vegetable consumption among all youth, a risk behaviour common to all four health behaviour clusters [62].

## Conclusions

Examining the patterns of obesity-related and other risky health behaviours, four subgroups of participants were identified in a large sample of youth from Ontario, Canada. Results reaffirm that not only do health behaviours co-occur, but they often do so in varying patterns, which can create challenges when designing public health interventions and population health prevention strategies. In this study, youth that belonged to all three of the clusters considered less healthy were at greater risk of being overweight/obese compared to youth with the healthiest behaviour patterns. To optimize limited prevention resources, it might be beneficial for public health interventions to target multiple modifiable risk factors that tend to cluster, tailored to particular subgroups of youth.

## Abbreviations

95% CI: 95% Confidence Interval
BMI: Body mass index
LCA: Latent class analysis
OR: Odds ratio
